# The Association Between Circulating Trans Fatty Acids and Thyroid Function Measures in U.S. Adults

**DOI:** 10.3389/fendo.2022.928730

**Published:** 2022-07-08

**Authors:** Xiaoqian Wang, Fengjuan Jiang, Wenqing Chen, Hui Yuan, Yuan Li

**Affiliations:** ^1^ Department of Clinical Nutrition, Nanjing Drum Tower Hospital, the Affiliated Hospital of Nanjing University Medical School, Nanjing, China; ^2^ Department of Urology, Nanjing Drum Tower Hospital, the Affiliated Hospital of Nanjing University Medical School, Nanjing, China

**Keywords:** trans fatty acids, thyroid hormone, TSH, NHANES, homeostasis

## Abstract

**Background:**

There has been controversial evidence regarding the effect of trans fatty acids (TFAs) on thyroid function in animal studies, and the epidemiological studies are lacking. We aimed to investigate the potential associations between circulating TFAs and thyroid function biomarkers in a U.S. adult population sample.

**Methods:**

We performed a cross-sectional survey with 626 adults aged ≥20 years who participated in the National Health and Nutrition Examination Survey (NHANES) 2009–2010. Multivariable linear regression models were constructed to elucidate the relationships between circulating concentrations of TFAs (palmitelaidic acid, vaccenic acid, elaidic acid, linoelaidic acid and the sum of the four TFAs) and a panel of thyroid function measures.

**Results:**

For 626 adults, positive associations were found between palmitelaidic acid, elaidic acid and total thyroxine (TT4), between palmitelaidic acid and total triiodothyronine (TT3), and between linolelaidic acid and thyroid stimulating hormone (TSH), while linolelaidic acid was negatively associated with free thyroxine (FT4) (all P<0.05). Besides, the four TFAs and the sum TFAs were positively associated with free triiodothyronine (FT3). Vaccenic acid, elaidic acid, linoelaidic acid and the sum TFAs were positively associated with FT3/FT4, while the four TFAs and the sum TFAs were negatively associated with FT4/TT4 (all P<0.05). In stratified analysis, the associations between thyroid function measures and the ratios remained significant in female. For men, linolelaidic acid was negatively associated with FT4 and elaidic acid and the sum TFAs were positively associated with FT3. Furthermore, the associations between TFAs and FT3/FT4 remained significant.

**Conclusion:**

Our findings revealed that TFAs exposure was associated with serum biomarkers of thyroid function. More researches are needed to evaluate the long-term health outcomes of these findings.

## Introduction

Trans Fatty acids (TFAs) are unsaturated fatty acids that contain one or more double bonds in the trans configuration, including industrial TFAs sourced from the process of partial hydrogenation of vegetable oils and ruminant TFAs formed by enzymes during hydrogenation in the rumen of animals (1). People are exposed to TFAs primarily through dietary intake of industry-processed high-fat foods (eg, burgers, fries, chicken cutlets, biscuits, cream) as well as animal products (eg, meat and dairy) (2). In fact, vaccenic acid (C18:1n-7t) and elaidic acid (C18:1n-9t) account for more than 80% of TFAs. It has been well established that high exposure of industrial TFAs is associated with the occurrence of various diseases, such as type 2 diabetes, mellitus, reproductive disease, total mortality, cardiovascular diseases (3–7).

Thyroid hormones are essential for energy metabolism and many physiological processes. The hypothalamus–pituitary–thyroid (HPT) axis regulates thyroid function by producing thyroxine (T4), triiodothyronine (T3), and thyroid stimulating hormone (TSH) (8). Numerous studies have shown that abnormal thyroid hormone variation had a detrimental impact on human health, such as an increased risk of incident chronic kidney disease and metabolic syndrome (9, 10). Recent years, researches have begun to explore the impact of diet on thyroid function. Stavroula found that thyroid function was negatively associated with non-homemade food, such as fast food and snacks (11). Besides, epidemiological data revealed that higher T4 and TSH level were observed for man adherence to pro-inflammatory diet (12). Animals and *in vitro* studies have observed that exposure to TFAs may influence thyroid hormone signaling (13–15). To our knowledge, studies about the potential influence of TFAs on thyroid function is relatively little. Moreover, epidemiological studies on the impact of TFAs on thyroid function have not been conducted.

The present analysis was carried out to investigate whether TFAs are associated with thyroid function measures in human. These results will help to further understand the biologic effects of TFAs on thyroid function, and provide a reference for consumption of TFAs in daily diet.

## Methods and Materials

### Study Population

All the data were from NHANES (2009–2010), which is a nationally cross-sectional study designed to evaluate the U.S. citizens’ health and nutritional status. The National Center for Health Statistics (NCHS) Ethics Review Board granted the approval of the study protocol and all the participants signed the informed consents. The adults with complete data on plasma TFAs and thyroid measures were eligible (n=808). We further excluded persons who were taking thyroid medications, had thyroid disease, or pregnant (16, 17). Finally, 626 adults were included in our analyses (participant flow chart, Line **Figure S1**).

### Plasma Thyroid Function Measurement

The 2009–2010 NHANES thyroid function measures contained total T4 (TT4), free T4 (FT4), total T3 (TT3), free T3 (FT3), TSH, thyroglobulin (Tg), thyroglobulin antibodies (TgAb) and thyroid peroxidase antibodies (TPOAb). We also calculated FT3/FT4, TT4/TT3, FT4/TT4, and FT3/TT3 based on the thyroid function measures, which reflect peripheral thyroid hormone metabolism (18).

Thyroid blood specimens were processed, stored and shipped to University of Washington, Seattle, WA. A paramagnetic particle, immunoenzymatic assay was applied for the quantitative determination of TT4, FT4, TT3 and FT3 in human plasma (19). Microparticle enzyme immunoassay was applied to detect serum TSH concentration. Tg was measured by a simultaneous one-step “sandwich” assay, while TPOAb and TgAb assay was a sequential two-step immunoenzymatic “sandwich” assay. The results of those thyroid function measures in the sample are determined from a stored, multi-point calibration curve and all meet the division’s quality control and quality assurance (QA/QC) performance criteria for accuracy and precision. Detailed information of the laboratory methods and QA/QC procedures can be viewed in the supplementary material (**Table S1**).

### Trans-Fatty Acids Measurement

Blood samples were drawn from participants’ veins in morning fasting following a standardized protocol and the serum was separated, stored, and frozen at −80°C. Gas chromatography coupled with mass spectrometry (GC/MS) was used to measure the total (free and esterified) content of four TFAs levels in the plasma samples: palmitelaidic acid (C16:1n-7t), vaccenic acid (C18:1n-7t), elaidic acid (C18:1n-9t), and linoelaidic acid (C18:2n-6t, 9t). The measurement method described by Lagerstedt et al. (20). Quality control procedures for all analyses followed a comprehensive data quality assurance program. Detailed description of laboratory methodology and QA/QC are available in the supplementary material (**Table S1**). Besides, the sum of TFAs was calculated by the following formula: Sum TFAs = palmitelaidic acid + vaccenic acid + elaidic acid + linoelaidic acid.

### Covariates

Sociodemographic data were collected by self-report. The following were incorporated as potential covariates: age, sex, Body mass index (BMI), education level, race/ethnicity, annual family income, urine iodine concentration (UIC), alcohol use and smoking status. Three age categories were: 20 to <40, 40 to <60 and ≥60 years. Education attainment groups: less than high school; high school/GED; more than high school. Race/ethnicity was categorized as Mexican American, Other Hispanic, Non-Hispanic White, Non-Hispanic Black and Other race. BMI was grouped by the following cut-offs <18.5, 18.5–24.9, 25–29.9 and ≥30 kg/m^2^. Two annual family income groups were < 20000 and ≥20000$. We divided UIC into three groups: deficient (UIC < 100 µg/L), normal (100–299 µg/L), excessive (≥ 300 µg/L) (21). There were three smoking status: current smoker (smokers or passive smokers when investigated), former smoker and nonsmoker. Alcohol was classified as yes (≥ 12 servings of any type of alcoholic beverage in any 1 year) and no.

### Statistical Analyses

Statistical analyses were performed by SPSS 22.0 for Windows (SPSS Inc.). First, the differences in serum thyroid function measurs in population subgroups were examined using One-way ANOVA or Kruskal-Wallis test. Then, the associations between each TFAs and serum thyroid function measures and ratios of thyroid function measures were demonstrated by multivariable linear regression model. Covariates were selected based on the results of population subgroups analysis (**Table S2**) and previous studies on the effect of environmental chemicals on thyroid function measures (22, 23). So, the regression model was adjusted by sex (dichotomous variable), age (continuous), BMI (continuous), race (categorical), UIC (continuous), alcohol and smoking (categorical). Besides, all plasma TFAs and thyroid function data were log-transformed because of non-normal distribution. Finally, stratification analyses were performed to explore whether sex could confound the relationship between plasma TFAs and thyroid function measures and the ratios of thyroid function measures. The level of significance in the study was 0.05 (P < 0.05).

## Results

### Demographic Characteristics

Population characteristics of 626 participants are presented in **Table 1**. 336 (53.7%) participants in our study were men. 44.4% were non-Hispanic white, while 17.3% were Non-Hispanic Black, 22.0% were Mexican American, and 4.0% were Other Race. 47.0% were in the category above high school. There were 434 (69.3%) participants being overweight or obese. According to self-report, 21.1% of participants were current smokers and 75.9% participants’ annual family income were above 20000$. Nearly half of the participants’ UIC were normal, while 36.9% were deficient and 13.6% were excessive.

**Table 1:** Distribution of demographic and laboratory characteristics.

Population Characteristics	N (%)
Sex	
Male	336 (53.7)
Female	290 (46.3)
Age category	
20-39	216 (34.5)
40-59	216 (34.5)
≥60	194 (31.0)
Race category	
Mexican American	138 (22.0)
Other Hispanic	77 (12.3)
Non-Hispanic White	278 (44.4)
Non-Hispanic Black	108 (17.3)
Other race	25 (4.0)
BMI category	
Underweight	5 (0.8)
Normal weight	187 (29.9)
Overweight	222 (35.4)
Obesity	212 (33.9)
Education category	
Less than high school	197 (31.5)
High school/GED	135 (21.5)
More than high school	294 (47.0)
Income	
<20000$	151 (24.1)
≥20000$	475 (75.9)
Smoking category	
Current smoker	132 (21.1)
Non-smoker	334 (53.3)
Former smoker	160 (25.6)
Alcohol category	
Yes	552 (88.2)
No	74 (11.8)
UIC	
Iodine deficient	231 (36.9)
Normal	310 (49.5)
Excessive iodine intake	85 (13.6)

BMI, body mass index; GED, General Equivalency Diploma; UIC, urine iodine concentration.

### Thyroid Function Measures

The **Table 2** presented the concentrations of thyroid function measures. The plasma mean concentration of TT4, FT4, TT3, FT3, TSH, Tg, TgAb and TPOAb levels were 0.80 ug/dL, 117.14 ng/dL, 3.24 ng/dL, 1.96 pg/mL, 14.14 mIU/L, 9.58 ug/L, 24.38 IU/mL and 8.02 IU/mL respectively. The distributions of thyroid function measures of different subgroups were shown in **Table S2**. Serum TT4, TT3, FT3 and TSH levels were significantly different in subgroup of age (all *P <0.05*). The subgroup of age above 60 had the highest TT4 (8.23 ug/dL) and TSH (2.24 mIU/L) levels and the lowest TT3 (111.38 ng/dL) and FT3 (3.07 pg/mL) concentrations. Except FT4 and TT3, the differences of thyroid function measures levels among sex subgroups were statistically significant (all *P <0.05*), and female group had higher serum TT4, TSH, Tg, TgAb and TPOAb levels. The thyroid function measures (TT4, TT3, FT3, TSH, Tg) levels were also significantly different among race subgroups (*P*=0.011, *P*=0.041, *P*=0.005, *P*=0.001 and *P*=1.82×10^-8^, respectively), Non-Hispanic White group had the highest TSH (2.17 mIU/L) and the lowest TT4 (7.88 ug/dL), TT3 (115.47 ng/dL) and FT3 (3.18 pg/mL) concentrations. Besides, in education categories, subgroup of more than high school had the lower TT4, TT3 and FT3 (all *P <0.05*). Additionally, the TT4 levels were significantly different in alcohol categories (*P*=0.20×10^-5^), and FT3 levels were significant different in smoking categories (*P*=0.018). Furthermore, compared to normal and excessive iodine intake group, iodine deficient group tended to have a higher TT4 (8.18 ug/dL) and Tg (16.30 ug/L) levels.

**Table 2:** Distribution of serum TFAs concentrations and thyroid function measures.

Serum Analyte	Mean	GM
Thyroid Function Measures	8.02	7.90
TT4 (ug/dL)	0.80	0.79
FT4 (ng/dL)	117.14	115.27
TT3 (ng/dL)	3.24	3.22
FT3 (pg/mL)	1.96	1.66
TSH (mIU/L)	14.14	9.42
Tg (ug/L)	9.58	0.82
TgAb (IU/mL)	24.38	1.23
TPOAb (IU/mL)	8.02	7.90
TFAs Exposure Biomarkers (umol/L)	0.80	0.79
Palmitelaidic acid	4.28	3.89
Elaidic acid	16.17	14.10
Vaccenic acid	21.07	18.50
Linolelaidic acid	1.78	1.61
Sum TFAs	43.29	38.62

TT4, total thyroxine; TT3, total triiodothyronine; FT4, free thyroxine; FT3, free triiodothyronine; TSH, thyroid stimulating hormone; Tg, thyroglobulin; TgAb, thyroglobulin antibodies; TPOAb, thyroid peroxidase antibodies; TFAs, trans fatty acids; GM, geometric mean.

### Trans Fatty Acids Level

The **Table 2** presented the concentrations of palmitelaidic acid, elaidic acid, vaccenic acid, linoelaidic acid and the sum of these TFAs respectively. The plasma mean concentration of vaccenic acid (21.07 umol/L) was the highest, followed by elaidic acid (16.17 umol/L), palmitelaidic acid (4.28 umol/L) and linolelaidic acid(1.78 umol/L). In addition, the mean level of sum TFAs was 43.29 umol/L.

### Associations Between TFAs and Thyroid Function Measures

The associations between log-transformed TFAs and thyroid function measures are presented in **Table 3**. Multivariable linear regression analysis showed that palmitelaidic acid and elaidic acid were significantly associated with increased TT4 (β = 0.021, 95% CI: 0.029–0.094, *P <0.05*; β = 0.035, 95% CI: 0.006–0.063, *P <0.05*, respectively), while linolelaidic acid was negatively correlated with FT4 (β = -0.067, 95% CI: -0.097– -0.036, *P <0.05*). Our results also revealed that all the four TFAs and the sum TFAs were positively associated with FT3 (β = 0.023, 95% CI: 0.004–0.042; β = 0.030, 95% CI: 0.014–0.047; β = 0.022, 95% CI: 0.006–0.039; β = 0.026, 95% CI: 0.007– 0.045; β = 0.029, 95% CI: 0.011– 0.047, respectively), while only palmitelaidic acid was positively correlated with TT3 (β = 0.038, 95% CI: 0.005–0.071, *P <0.05*). Additionally, the analysis results showed that only plasma linolelaidic acid was significantly correlated with plasma TSH (β = 0.124, 95% CI: 0.013–0.236, *P <0.05*). Furthermore, there was no significantly association between Tg, TgAb and TPOAb and TFAs in our study.

**Table 3:** Multivariate regression analyses of log-transformed serum TFAs in relation to log-transformed serum thyroid function measures.

	TT4(ug/dL)	FT4(ng/dL)	TT3(ng/dL)	FT3(pg/mL)	TSH(mIU/L)	Tg(ug/L)	TgAb(IU/mL)	TPOAb (IU/mL)
β (95% CI)								
Palmitelaidic acid	0.061 (0.029,0.094)*	-0.004 (-0.035,0.027)	0.038 (0.005,0.071)*	0.023 (0.004,0.042)*	0.060 (-0.053,0.173)	-0.115 (-0.302,0.072)	0.210 (0.011,0.409)	-0.192 (0.549,0.164)
Elaidic acid	0.035 (0.006,0.063)*	-0.022 (-0.049,0.005)	0.024 (-0.005,0.053)	0.030 (0.014,0.047)*	0.049 (-0.050,0.147)	0.040 (-0.123,0.203)	0.077 (-0.097,0.251)	-0.025 (0.336,0.287)
Vaccenic acid	0.019 (-0.009,0.047)	-0.020 (-0.046,0.007)	0.018 (-0.010,0.047)	0.022 (0.006,0.039)*	0.070 (-0.027,0.167)	-0.044 (-0.204,0.117)	0.125 (-0.046,0.296)	-0.077 (0.384,0.229)
Linolelaidic acid	-0.013 (-0.045,0.020)	-0.067 (-0.097,0.036)*	0.017 (-0.016,0.050)	0.026 (0.007,0.045)*	0.124 (0.013,0.236)*	0.033 (-0.152,0.218)	0.098 (-0.099,0.295)	-0.071 (0.424,0.282)
Sum TFAs	0.030 (-0.001,0.061)	-0.025 (-0.054,0.005)	0.025 (-0.006,0.056)	0.029 (0.011,0.047)*	0.077 (-0.030,0.181)	-0.025 (-0.202,0.151)	0.138 (-0.050,0.325)	-0.063 (0.399,0.274)

TT4, total thyroxine; TT3, total triiodothyronine; FT4, free thyroxine; FT3, free triiodothyronine; TSH, thyroid stimulating hormone; Tg, thyroglobulin; TgAb, thyroglobulin antibodies; TPOAb, thyroid peroxidase antibodies; CI, confidence interval; *P < 0.05; Model was adjusted by sex category, age, BMI, race category, UIC, alcohol category and smoking category.

### Associations Between TFAs and the Ratios of Thyroid Function Measures

The associations between log-transformed TFAs and the ratios of thyroid function measures are shown in **Table 4**. The multivariate linear regression model was adjusted by sex, age, BMI, race, UIC, alcohol use and tobacco smoking. We found that the associations between elaidic acid, vaccenic acid, linoelaidic acid and the sum TFAs and FT3/FT4 were statistically significant (β = 0.491, 95% CI: 0.195–0.786, *P <0.05*; β = 0.408, 95% CI: 0.116–0.700, *P <0.05*; β = 0.873, 95% CI: 0.541–1.204, *P <0.05*; β = 0.519, 95% CI: 0.199–0.839, *P <0.05*, respectively). Besides, the significantly inverse associations between all the four TFAs and the sum TFAs and FT4/TT4 were observed.

**Table 4:** Multivariate regression analyses of log-transformed serum TFAs in relation to ratios of thyroid function measures.

	FT3/FT4	TT4/TT3	FT4/TT4	FT3/TT3
β (95% CI)				
Palmitelaidic acid	0.260 (-0.082,0.601)	0.004 (-0.003,0.010)	-0.015 (-0.022,-0.008) *	-0.001 (-0.003,0.001)
Elaidic acid	0.491 (0.195,0.786) *	0.002 (-0.004,0.008)	-0.013 (-0.019,-0.006) *	0.001 (-0.001,0.002)
Vaccenic acid	0.408 (0.116,0.700) *	2.41×10^-4^ (-0.005,0.006)	-0.009 (-0.015,-0.003) *	4.35×10^-4^ (-0.001,0.002)
Linolelaidic acid	0.873 (0.541,1.204) *	-0.005 (-0.011,0.001)	-0.012 (-0.019,-0.004) *	0.001 (-0.001,0.003)
Sum TFAs	0.519 (0.199,0.839) *	0.001 (-0.005,0.007)	-0.013 (-0.019,-0.006) *	0.001 (-0.001,0.002)

FT3, free triiodothyronine; FT4, free thyroxine; TT4, total thyroxine; TT3, total triiodothyronine; CI, confidence interval; *P < 0.05; Model was adjusted by sex category, age, BMI, race category, UIC, alcohol category and smoking category.

### Stratified Analysis of Correlations Between Plasma TFAs and Thyroid Function Measures and the Corresponding Ratios

To further explore whether sex could confound the relationship between plasma TFAs and thyroid function measures and the ratios, stratification analyses were performed based on sex categories (**Tables S3**, **S4**). The associations between TFAs and thyroid function measures and the ratios shown in **Tables 3**, **4** remained significant in female. In addition, stratification analyses showed positive associations between sum TFAs and TT4, and between elaidic acid, linolelaidic acid and sum TFAs and TT3 in female subgroup. For male, linolelaidic acid was negatively correlated with FT4 (β = -0.057, 95% CI: -0.098– -0.016, P <0.05) and elaidic acid and the sum TFAs was positively associated with FT3. Furthermore, the associations between TFAs and FT3/FT4 shown in **Table 4** remained significant, while there was no significant association between TFAs and FT4/TT4 in male (**Table S4**).

## Discussion

In this study, we observed positive associations between TFAs and plasma TT4, TT3, FT3, TSH and FT3/FT4 ratio, and negative associations between TFAs and FT4 and FT4/TT4. Additionally, sex-stratified subgroup analysis showed that the associations between thyroid function measures and the ratios remained significant in female. Besides, the stratified analysis showed the inconsistent associations of TFAs and thyroid function measures for different gender.

Under normal physiological situation, the HPT axis is tightly regulated by a homeostatic feedback loop. However, illnesses or exposure to environmental contaminants may perturb the regulatory system. At present, limited data are available on the potential impact of TFAs on thyroid function. Our research revealed that TFAs were associated with TT4 and TT3 concentrations, which is consistent with the previous animal experiments (13–15). However, no changed TSH level were observed by infusion fat mixtures high in TFAs for three weeks in lactating cows (13). Furthermore, research has confirmed that FT3/FT4 ratio is a valid index of deiodinase activity which converting FT4 into FT3 (24). Our findings showed positive associations between TFAs and deiodinase activity, while the animal study found that TFAs can inhibit 5’-deiodinase activity *in vitro*. One possible explanation for the inconsistency in animal experiments may be the methodological and species differences. In the study of human populations, the consumption of non-homemade food and pro-inflammatory diet was positively correlated with TT4 and TSH level (11, 12), but there is no studies on the impact of TFAs on thyroid function.

The mechanisms underlying the associations are not yet clear. Growing evidence suggests that inflammation is linked with abnormal thyroid function (25–27). The secretion of thyroid hormones might be inhibited in intermediate levels of pro-inflammatory markers and potentiated in low levels (28). TFAs, a definite pro-inflammatory food parameter, induced low-grade inflammation, which may interpret the positive associations between TFAs and serum TT4, TT3, FT3 and TSH. As part of HPT axis homeostatic feedback loop, 5’-deiodinase activity is induced at the transcriptional level by elevated circulating FT3 and converts more FT4 to FT3. So, we also observed the positive association between TFAs and FT3/FT4 which reflects the 5’-deiodinase activity. Besides, pro-inflammatory cytokines have the effect on synthesis and activity of 5’-deiodinase (29). Taken together, more in-depth studies are required to verify the probable sites and mechanism on human.

Furthermore, we observed the relationships of the plasma TFAs and thyroid function measures and corresponding ratios were remained significant in female. Many studies show that women are more susceptible to thyroid disease than men, which may partially be caused by the increased requirements on the thyroid during pregnancy and lactation, or the impact of estrogens on thyroid function (30, 31). Thus, we inferred that our findings may be due to the fact that female HPT axis homeostasis is more susceptible to TFAs.

The study is the first cross-sectional population research to examine the relationship between serum TFAs and thyroid function measures in US adults. Serum levels of TFAs subtypes reflect the availability in the diet because human do not synthesize TFAs and the direct exposure assessments circumvents the recall bias of food frequency questionnaires. However, there were some limitations in the present study. Firstly, the plasma levels of TFAs and thyroid hormones were examined only for a single measurement, which failed to reflect the long-term plasma thyroid index status (32, 33). Secondly, the cross-sectional analysis cannot determine the causation. Dietary TFAs are absorbed, activated, oxidized, and acylated into ester lipids much like saturated fatty acids *in vivo*, and HPT axis is involved in this process (34) There may be dual-direction effects between TFAs and thyroid hormones. Finally, the data on pediatric and adolescent are lacking in our study. Further researches of populations at various life stages are warranted in this area.

## Conclusion

In conclusion, the current study suggests that circulating TFAs are associated with levels of thyroid function measures in adults, indicting a potential influence of TFAs on the thyroid function. However, prospective work is required to clarify the underlying mechanisms.

Supplementary Materials: Line **Figure S1**: Flow chart of study population exclusion criteria; **Table S1**: The detailed websites of laboratory methods and QA/QC procedures; **Table S2**: Distribution of thyroid function measures. **Table S3**: Stratified analysis of correlations between log-transformed serum trans fatty acids and log-transformed thyroid function measures by sex category; **Table S4**: Stratified analysis of correlations between log-transformed serum trans fatty acids and ratios of thyroid function measures by sex category.

## Data Availability Statement

The datasets presented in this study can be found in online repositories. The names of the repository/repositories and accession number(s) can be found below: The data used to support the findings of this study are available from ‘NHANES’ database (https://www.cdc.gov/nchs/nhanes/index.htm).

## Ethics Statement

The studies involving human participants were reviewed and approved by The National Center for Health Statistics (NCHS) Ethics Review Board granted the approval of the study protocol. The patients/participants provided their written informed consent to participate in this study.

## Author Contributions

XW and FJ were involved in the study design and drafted the manuscript. HY participated in data acquisition and statistical analysis. WC contributed to re-analysis the data. YL was responsible for manuscript revision. All authors read and approved the final manuscript. All authors contributed to the article and approved the submitted version.

## Funding

This research was funded by Nanjing Medical Science and Technology Development, grant number YKK18079.

## Conflict of Interest

The authors declare that the research was conducted in the absence of any commercial or financial relationships that could be construed as a potential conflict of interest.

## Publisher’s Note

All claims expressed in this article are solely those of the authors and do not necessarily represent those of their affiliated organizations, or those of the publisher, the editors and the reviewers. Any product that may be evaluated in this article, or claim that may be made by its manufacturer, is not guaranteed or endorsed by the publisher.

## References

[1] Lichtenstein AH. Dietary Trans Fatty Acids and Cardiovascular Disease Risk: Past and Present. Curr Atheroscler Rep (2014) 16(8):433. doi: 10.1007/s11883-014-0433-1 24907053

[2] Wanders AJ Zock PL Brouwer IA. Trans Fat Intake and Its Dietary Sources in General Populations Worldwide: A Systematic Review. Nutrients. (2017) 9(8):840. doi: 10.3390/nu9080840 PMC5579633 28783062

[3] Calder PC. Functional Roles of Fatty Acids and Their Effects on Human Health. JPEN J parenteral enteral Nutr (2015) 39(Suppl 1):18s–32s. doi: 10.1177/0148607115595980 26177664

[4] Zhao Z Jiang Y Li Q Cai Y Yin H Zhang L. Spatial Correlation Analysis of Polycyclic Aromatic Hydrocarbons (PAHs) and Organochlorine Pesticides (OCPs) in Sediments Between Taihu Lake and its Tributary Rivers. Ecotoxicol Environ Safety (2017) 142:117–28. doi: 10.1016/j.ecoenv.2017.03.039 28395204

[5] Hanis T Zidek V Sachova J Klir P Deyl Z. Effects of Dietary Trans-Fatty Acids on Reproductive Performance of Wistar Rats. Br J Nutr (1989) 61(3):519–29. doi: 10.1079/BJN19890140 2758008

[6] Bhathena SJ. Relationship Between Fatty Acids and the Endocrine and Neuroendocrine System. Nutr Neurosci (2006) 9(1-2):1–10. doi: 10.1080/10284150600627128 16910164

[7] Wang Q Imamura F Lemaitre RN Rimm EB Wang M King IB. Plasma Phospholipid Trans-Fatty Acids Levels, Cardiovascular Diseases, and Total Mortality: The Cardiovascular Health Study. J Am Heart Assoc (2014) 3(4):e000914. doi: 10.1161/JAHA.114.000914 25164946 PMC4310377

[8] Stathatos N. Thyroid Physiology. Med Clin North Am (2012) 96(2):165–73. doi: 10.1016/j.mcna.2012.01.007 22443969

[9] Zhang Y Chang Y Ryu S Cho J Lee WY Rhee EJ. Thyroid Hormone Levels and Incident Chronic Kidney Disease in Euthyroid Individuals: The Kangbuk Samsung Health Study. Int J Epidemiol (2014) 43(5):1624–32. doi: 10.1093/ije/dyu126 25011453

[10] Kim HJ Bae JC Park HK Byun DW Suh K Yoo MH. Association of Triiodothyronine Levels With Future Development of Metabolic Syndrome in Euthyroid Middle-Aged Subjects: A 6-Year Retrospective Longitudinal Study. Eur J Endocrinol (2017) 176(4):443–52. doi: 10.1530/EJE-16-0734 28100631

[11] Lambrinakou S Katsa ME Zyga S Ioannidis A Sachlas A Panoutsopoulos G. Correlations Between Nutrition Habits, Anxiety and Metabolic Parameters in Greek Healthy Adults. Adv Exp Med Biol (2017) 987:23–34. doi: 10.1007/978-3-319-57379-3_3 28971444

[12] Liu N Ma F Feng Y Ma X. The Association Between the Dietary Inflammatory Index and Thyroid Function in U.S. Adult Males Nutrients (2021) 13(10):3330. doi: 10.3390/nu13103330 34684331 PMC8540204

[13] Romo GA Elsasser TH Kahl S Erdman RA Casper DP. Dietary Fatty Acids Modulate Hormone Responses in Lactating Cows: Mechanistic Role for 5'-Deiodinase Activity in Tissue. Domest Anim Endocrinol (1997) 14(6):409–20. doi: 10.1016/S0739-7240(97)00046-5 9437577

[14] Corino C Pastorelli G Rosi F Bontempo V Rossi R. Effect of Dietary Conjugated Linoleic Acid Supplementation in Sows on Performance and Immunoglobulin Concentration in Piglets. J Anim Sci (2009) 87(7):2299–305. doi: 10.2527/jas.2008-1232 19286821

[15] Sechman A Pieszka M Rzasa J Migdal W Wojtysiak D Pustkowiak H. The Effect of Dietary Conjugated Linoleic Acid on the Levels of Lipids, Cholesterol and Iodothyronines in the Blood of Pigs. J Anim Feed Sci (2007) 16(2):193–204. doi: 10.22358/jafs/66738/2007

[16] Turyk ME Anderson HA Persky VW. Relationships of Thyroid Hormones With Polychlorinated Biphenyls, Dioxins, Furans, and DDE in Adults. Environ Health Perspect (2007) 115(8):1197–203. doi: 10.1289/ehp.10179 PMC1940071 17687447

[17] Van Gerwen M Alpert N Alsen M Ziadkhanpour K Taioli E Genden E. The Impact of Smoking on the Association Between Perfluoroalkyl Acids (PFAS) and Thyroid Hormones: A National Health and Nutrition Examination Survey Analysis. Toxics (2020) 8(4):116. doi: 10.3390/toxics8040116 PMC7768414 33316920

[18] Braathen M Derocher AE Wiig Ø Sørmo EG Lie E Skaare JU. Relationships Between PCBs and Thyroid Hormones and Retinol in Female and Male Polar Bears. Environ Health Perspect (2004) 112(8):826–33. doi: 10.1289/ehp.6809 PMC1242008 15175168

[19] Webster GM Rauch SA Marie NS Mattman A Lanphear BP Venners SA. Cross-Sectional Associations of Serum Perfluoroalkyl Acids and Thyroid Hormones in U.S. Adults: Variation According to TPOAb and Iodine Status (NHANES 2007-2008). Environ Health Perspect (2016) 124(7):935–42. doi: 10.1289/ehp.1409589 PMC4937851 26517287

[20] Lagerstedt SA Hinrichs DR Batt SM Magera MJ Rinaldo P McConnell JP. Quantitative Determination of Plasma C8-C26 Total Fatty Acids for the Biochemical Diagnosis of Nutritional and Metabolic Disorders. Mol Genet Metab (2001) 73(1):38–45. doi: 10.1006/mgme.2001.3170 11350181

[21] Park S Kim WG Jeon MJ Kim M Oh HS Han M. Serum Thyroid-Stimulating Hormone Levels and Smoking Status: Data From the Korean National Health and Nutrition Examination Survey Vi. Clin Endocrinol (Oxf) (2018) 88(6):969–76. doi: 10.1111/cen.13606 29604104

[22] Meeker JD Ferguson KK. Relationship Between Urinary Phthalate and Bisphenol A Concentrations and Serum Thyroid Measures in U.S. Adults and Adolescents From the National Health and Nutrition Examination Survey (NHANES) 2007-2008. Environ Health Perspect (2011) 119(10):1396–402. doi: 10.1289/ehp.1103582 PMC3230451 21749963

[23] Fortenberry GZ Hu H Turyk M Barr DB Meeker JD. Association Between Urinary 3, 5, 6-Trichloro-2-Pyridinol, a Metabolite of Chlorpyrifos and Chlorpyrifos-Methyl, and Serum T4 and TSH in NHANES 1999-2002. Sci Total Environ (2012) 424:351–5. doi: 10.1016/j.scitotenv.2012.02.039 PMC3327766 22425279

[24] Yoshimura Noh J Momotani N Fukada S Ito K Miyauchi A Amino N. Ratio of Serum Free Triiodothyronine to Free Thyroxine in Graves' Hyperthyroidism and Thyrotoxicosis Caused by Painless Thyroiditis. Endocr J (2005) 52(5):537–42. doi: 10.1507/endocrj.52.537 16284430

[25] Malyszko J Malyszko JS Pawlak K Mysliwiec M. Thyroid Function, Endothelium, and Inflammation in Hemodialyzed Patients: Possible Relations? J Ren Nutr (2007) 17(1):30–7. doi: 10.1053/j.jrn.2006.07.003 17198929

[26] Liu XZ Wang JM Ji YX Zhao DB. Monocyte-To-High-Density Lipoprotein Cholesterol Ratio is Associated With the Presence and Size of Thyroid Nodule Irrespective of the Gender. Lipids Health Dis (2020) 19(1):36. doi: 10.1186/s12944-020-1196-z 32164741 PMC7069177

[27] Grubeck-Loebenstein B Buchan G Chantry D Kassal H Londei M Pirich K. Analysis of Intrathyroidal Cytokine Production in Thyroid Autoimmune Disease: Thyroid Follicular Cells Produce Interleukin-1 Alpha and Interleukin-6. Clin Exp Immunol (1989) 77(3):324–30. PMC1542067 2680182

[28] Bendtzen K Buschard K Diamant M Horn T Svenson M. Possible Role of IL-1, TNF-Alpha, and IL-6 in Insulin-Dependent Diabetes Mellitus and Autoimmune Thyroid Disease. Thyroid Cell Group Lymphokine Res (1989) 8(3):335–40. 2674559

[29] Yu J Koenig RJ. Regulation of Hepatocyte Thyroxine 5'-Deiodinase by T3 and Nuclear Receptor Coactivators as a Model of the Sick Euthyroid Syndrome. J Biol Chem (2000) 275(49):38296–301. doi: 10.1074/jbc.M004866200 10995750

[30] Laurberg P Nøhr SB Pedersen KM Hreidarsson AB Andersen S Bülow Pedersen I. Thyroid Disorders in Mild Iodine Deficiency. Thyroid. (2000) 10(11):951–63. doi: 10.1089/thy.2000.10.951 11128722

[31] Furlanetto TW Nguyen LQ Jameson JL. Estradiol Increases Proliferation and Down-Regulates the Sodium/Iodide Symporter Gene in FRTL-5 Cells. Endocrinology. (1999) 140(12):5705–11. doi: 10.1210/endo.140.12.7197 10579335

[32] Katan MB Deslypere JP van Birgelen AP Penders M Zegwaard M. Kinetics of the Incorporation of Dietary Fatty Acids Into Serum Cholesteryl Esters, Erythrocyte Membranes, and Adipose Tissue: An 18-Month Controlled Study. J Lipid Res (1997) 38(10):2012–22. doi: 10.1016/S0022-2275(20)37132-7 9374124

[33] Surks MI Goswami G Daniels GH. The Thyrotropin Reference Range Should Remain Unchanged. J Clin Endocrinol Metab (2005) 90(9):5489–96. doi: 10.1210/jc.2005-0170 16148346

[34] Kinsella JE Bruckner G Mai J Shimp J. Metabolism of Trans Fatty Acids With Emphasis on the Effects of Trans, Trans-Octadecadienoate on Lipid Composition, Essential Fatty Acid, and Prostaglandins: An Overview. Am J Clin Nutr (1981) 34(10):2307–18. doi: 10.1093/ajcn/34.10.2307 6794350

