# Supplementary material for: The Association Between Circulating Trans Fatty Acids and Thyroid Function Measures in U.S. Adults
Source: Front Endocrinol (Lausanne). 2022 Jul 8;13:928730. doi: 10.3389/fendo.2022.928730 (PMC9309269; doi:10.3389/fendo.2022.928730)
Supplement: Supplementary file 1 [file DataSheet_1.docx]

Analysed (n=626)

2462 adults were tested for four trans fatty acids

1654 were excluded due to missing data on thyroid function measures

808 adults with complete data on blood TFAs and thyroid function measures

182 were further excluded

86 reported a history of diagnosed thyroid disease

85 had taken prescription medications

11 were currently pregnant

Line Figure S1: Flow chart of study population exclusion criteria.

**Table S1.** The detailed websites of laboratory methods and quality control and quality assurance procedures.

| Analyte | website |
| --- | --- |
| TT4 | https://wwwn.cdc.gov/nchs/data/nhanes/2009-2010/labmethods/THYROD_F_met_TT4.pdf |
| FT4 | https://wwwn.cdc.gov/nchs/data/nhanes/2009-2010/labmethods/THYROD_F_met_FT4.pdf |
| TT3 | https://wwwn.cdc.gov/nchs/data/nhanes/2009-2010/labmethods/THYROD_F_met_TT3.pdf |
| FT3 | https://wwwn.cdc.gov/nchs/data/nhanes/2009-2010/labmethods/THYROD_F_met_FT3.pdf |
| TSH | https://wwwn.cdc.gov/nchs/data/nhanes/2009-2010/labmethods/THYROD_F_met_TSH.pdf |
| Tg | https://wwwn.cdc.gov/nchs/data/nhanes/2009-2010/labmethods/THYROD_F_met_Thyroglobin.pdf |
| TgAb | https://wwwn.cdc.gov/nchs/data/nhanes/2009-2010/labmethods/THYROD_F_met_Thyroglobulin%20Antibodies.pdf |
| TPOAb | https://wwwn.cdc.gov/nchs/data/nhanes/2009-2010/labmethods/THYROD_F_met_Thyroid_Peroxidase_Antibodies.pdf |
| TFAs | https://wwwn.cdc.gov/nchs/data/nhanes/2009-2010/labmethods/TFA_F_MET.pdf |

Note: TT4, total thyroxine; TT3, total triiodothyronine; FT4, free thyroxine; FT3, free triiodothyronine; TSH, thyroid stimulating hormone; Tg, thyroglobulin; TgAb, thyroglobulin antibodies; TPOAb, thyroid peroxidase antibodies; TFAs, trans fatty acids..

**Table S2.** Distribution of thyroid function measures.

| Variables | TT4  (ug/dL) | FT4  (ng/dL) | TT3  (ng/dL) | FT3  (pg/mL) | TSH  (mIU/L) | Tg  (ug/L) | TgAb  (IU/mL) | TPOAb (IU/mL) |
| --- | --- | --- | --- | --- | --- | --- | --- | --- |
|  | Mean ± SD | | | | | | | |
| Sex |  |  |  |  |  |  |  |  |
| Male | 7.79±1.33* | 0.80±0.13 | 117.63±20.64 | 3.32±0.38* | 1.87±1.13* | 12.19±11.3* | 4.82±41.30* | 8.92±42.80* |
| Female | 8.29±1.46* | 0.79±0.13 | 116.59±21.68 | 3.15±0.35* | 2.08±1.22* | 16.40±18.5* | 15.14±112.9* | 42.42±137.9* |
| Age category |  |  |  |  |  |  |  |  |
| 20-39 | 8.00±1.39* | 0.80±0.13 | 123.61±21.3* | 3.42±0.38* | 1.85±1.19* | 12.01±9.98 | 5.86±63.20 | 16.58±74.18 |
| 40-59 | 7.85±1.38* | 0.79±0.13 | 116.86±20.7* | 3.22±0.34* | 1.83±1.07* | 15.25±16.87 | 1.53±6.03 | 36.48±130.26 |
| ≥60 | 8.23±1.46 | 0.80±0.13 | 111.38±19.7* | 3.07±0.33* | 2.24±1.24* | 15.17±17.83 | 22.53±131.17 | 19.61±84.93 |
| Race category |  |  |  |  |  |  |  |  |
| Mexican American | 8.33±1.47* | 0.79±0.13 | 120.13±20.1* | 3.32±0.38* | 1.79±1.1* | 9.21±7.64* | 1.70±4.16 | 25.04±107.83 |
| Other Hispanic | 7.98±1.24* | 0.79±0.12 | 120.05±17.7* | 3.28±0.38* | 2.00±1.1* | 14.45±16.8* | 33.73±181.49 | 42.14±148.78 |
| Non-Hispanic White | 7.88±1.35* | 0.80±0.13 | 115.47±21.1* | 3.18±0.36* | 2.17±1.2* | 14.30±13.8* | 4.48±43.43 | 20.52±82.27 |
| Non-Hispanic Black | 7.91±1.55* | 0.78±0.12 | 115.69±24.5* | 3.25±0.39* | 1.69±0.9* | 19.11±19.5* | 4.36±30.53 | 17.51±80.37 |
| Other race | 8.50±1.40* | 0.84±0.09 | 116.64±18.6* | 3.31±0.37* | 1.76±1.0* | 17.12±25.7* | 58.35±206.94 | 38.45±127.22 |
| BMI category |  |  |  |  |  |  |  |  |
| Underweight | 8.02±1.95 | 0.80±0.08 | 105.20±16.05 | 3.11±0.52 | 1.90±1.20 | 10.30±5.82 | 1.08±0.69 | 3.52±5.38 |
| Normal weight | 7.98±1.42 | 0.81±0.13 | 116.63±21.85 | 3.25±0.39 | 1.96±1.16 | 14.18±14.37 | 10.56±80.24 | 23.91±102.94 |
| Overweight | 7.89±1.44 | 0.79±0.13 | 116.28±21.21 | 3.22±0.34 | 1.92±1.12 | 14.09±17.45 | 13.19±107.77 | 30.35±118.18 |
| Obesity | 8.20±1.35 | 0.79±0.12 | 118.78±20.41 | 3.26±0.39 | 2.02±1.25 | 14.24±13.68 | 5.23±49.33 | 19.03±75.33 |
| Education category |  |  |  |  |  |  |  |  |
| Less than high school | 8.31±1.40* | 0.80±0.12 | 119.97±22.4* | 3.29±0.40* | 1.97±1.17 | 13.95±16.50 | 10.39±103.2* | 23.98±114.55 |
| High school/GED | 7.97±1.32* | 0.81±0.14 | 117.35±20.3* | 3.29±0.41* | 1.83±1.05 | 16.36±17.82 | 1.39±5.32* | 11.57±49.04 |
| More than high school | 7.85±1.44* | 0.79±0.13 | 115.15±20.3* | 3.19±0.34* | 2.02±1.23 | 13.24±12.88 | 12.75±85.65* | 30.51±106.72 |
| Income |  |  |  |  |  |  |  |  |
| <20000$ | 8.13±1.46 | 0.79±0.13 | 118.15±21.60 | 3.27±0.40 | 2.01±1.26 | 15.58±18.11 | 3.48±25.12 | 18.08±66.73 |
| ≥20000$ | 7.98±1.40 | 0.80±0.13 | 116.85±21.03 | 3.23±0.37 | 1.96±1.15 | 13.78±14.25 | 11.63±94.17 | 26.66±109.22 |
| Smoking category (n, %) |  |  |  |  |  |  |  |  |
| Current smoker | 7.94±1.41 | 0.79±0.13 | 119.48±24.77 | 3.30±0.41* | 1.80±1.14 | 15.78±14.80 | 11.82±122.63 | 26.04±113.54 |
| Non-smoker | 8.05±1.43 | 0.80±0.12 | 117.37±19.92 | 3.25±0.36* | 1.97±1.16 | 14.26±17.33 | 8.42±63.02 | 28.80±108.68 |
| Former smoker | 8.04±1.38 | 0.81±0.13 | 114.76±20.13 | 3.18±0.37* | 2.09±1.23 | 12.53±10.03 | 10.15±77.79 | 13.73±62.44 |
| Alcohol category (n, %) |  |  |  |  |  |  |  |  |
| Yes | 7.92±1.37* | 0.79±0.13 | 116.83±20.76 | 3.25±0.37 | 1.99±1.20 | 13.93±13.91 | 7.48±74.46 | 22.48±98.53 |
| No | 8.75±1.54* | 0.81±0.14 | 119.51±23.60 | 3.19±0.38 | 1.77±1.01 | 15.68±22.99 | 25.52±128.04 | 38.46±111.26 |
| UIC (n, %) |  |  |  |  |  |  |  |  |
| Iodine deficient | 8.18±1.40* | 0.80±0.13 | 119.21±21.23 | 3.28±0.35 | 1.95±1.24 | 16.30±17.4* | 15.81±121.32 | 31.58±119.17 |
| Normal | 7.98±1.39* | 0.79±0.13 | 116.10±19.57 | 3.22±0.37 | 1.93±1.05 | 13.53±14.8* | 6.89±53.23 | 20.99±86.01 |
| Excessive iodine intake | 7.76±1.49* | 0.81±0.13 | 115.33±25.59 | 3.25±0.45 | 2.11±1.40 | 10.49±7.94* | 2.69±10.90 | 17.09±90.81 |

Note: TT4, total thyroxine; TT3, total triiodothyronine; FT4, free thyroxine; FT3, free triiodothyronine; TSH, thyroid stimulating hormone; Tg, thyroglobulin; TgAb, thyroglobulin antibodies; TPOAb, thyroid peroxidase antibodies; SD, standard deviation; BMI, body mass index; GED, General Equivalency Diploma; UIC, urine iodine concentration. * P < 0.05 for a significant difference in thyroid function measurements in the category using weighted ANOVA or Kruskal-Wallis test.

**Table S3.** Stratified analysis of correlations between log-transformed serum trans fatty acids and log-transformed thyroid function measures by sex category.

|  | TT4 (ug/dL) | FT4 (ng/dL) | TT3 (ng/dL) | FT3 (pg/mL) | TSH (mIU/L) |
| --- | --- | --- | --- | --- | --- |
|  | β (95% CI) | | | | |
| Palmitelaidic acid |  |  |  |  |  |
| Male | 0.029 (-0.017,0.075) | -0.002 (-0.045,0.040) | 0.019 (-0.025,0.064) | 0.022 (-0.003,0.047) | 0.071 (-0.079,0.221) |
| Female | 0.097 (0.050,0.143) * | -0.007 (-0.052,0.039) | 0.066 (0.016,0.116)* | 0.032 (0.003,0.062)* | 0.055 (-0.119,0.230) |
| Elaidic acid |  |  |  |  |  |
| Male | 0.007 (-0.031,0.046) | -0.020 (-0.056,0.016) | 0.002 (-0.035,0.039) | 0.026 (0.005,0.047) * | 0.081 (-0.045,0.206) |
| Female | 0.068 (0.026,0.111) * | -0.029 (-0.071,0.012) | 0.055 (0.008,0.101) * | 0.038 (0.011,0.065)* | 0.014 (-0.145,0.174) |
| Vaccenic acid |  |  |  |  |  |
| Male | 2.12×10^-4^(-0.037,0.037) | -0.021 (-0.056,0.013) | 0.008 (-0.028,0.044) | 0.017 (-0.004,0.037) | 0.113 (-0.008,0.234) |
| Female | 0.043 (-0.001,0.086) | -0.020 (-0.063,0.022) | 0.034 (-0.012,0.081) | 0.031 (0.004,0.059)* | 0.013 (-0.149,0.174) |
| Linolelaidic acid |  |  |  |  |  |
| Male | -0.037 (-0.081,0.008) | -0.057(-0.098,-0.016)* | -0.021 (-0.064,0.022) | 0.020 (-0.004,0.045) | 0.073 (-0.072,0.218) |
| Female | 0.016 (-0.032,0.064) | -0.082(-0.127,-0.037)* | 0.067 (0.016,0.117) * | 0.033 (0.003,0.062) * | 0.188 (0.013,0.362)* |
| Sum TFAs |  |  |  |  |  |
| Male | 0.003 (-0.038,0.044) | -0.025 (-0.063,0.013) | 0.006 (-0.034,0.046) | 0.023 (0.001,0.046)* | 0.111 (-0.023,0.244) |
| Female | 0.065 (0.018,0.113) * | -0.029 (-0.075,0.016) | 0.054 (0.003,0.105)* | 0.040 (0.010,0.070)* | 0.030 (-0.146,0.206) |

Note: TT4, total thyroxine; TT3, total triiodothyronine; FT4, free thyroxine; FT3, free triiodothyronine; TSH, thyroid stimulating hormone; Tg, thyroglobulin; TgAb, thyroglobulin antibodies; TPOAb, thyroid peroxidase antibodies; CI, confidence interval; * P < 0.05; Model was adjusted by age, BMI, race category, UIC, alcohol category and smoking category.

**Table S4.** Stratified analysis of correlations between log-transformed serum trans fatty acids and ratios of thyroid function measures by sex category.

|  | FT3/FT4 | TT4/TT3 | FT4/TT4 | FT3/TT3 |
| --- | --- | --- | --- | --- |
|  | β (95% CI) | | | |
| Palmitelaidic acid |  |  |  |  |
| Male | 0.281 (-0.206,0.768) | 0.003 (-0.005,0.011) | -0.008 (-0.018,-0.002) | 4.16×10^-4^ (-0.002,0.003) |
| Female | 0.316 (-0.166,0.798) | 0.003 (-0.007,0.013) | -0.023 (-0.033,-0.012)* | -0.002 (-0.005,0.001) |
| Elaidic acid |  |  |  |  |
| Male | 0.460 (0.056,0.865) * | 0.002 (-0.005,0.009) | -0.007 (-0.015,-0.002) | 0.002 (0.000,0.004) |
| Female | 0.581 (0.144,1.018) * | 0.001 (-0.008,0.011) | -0.021 (-0.031,-0.012)* | -0.001 (-0.003,0.001) |
| Vaccenic acid |  |  |  |  |
| Male | 0.393 (0.000.0786) * | 5.46×10^-5^ (-0.007,0.007) | -0.006 (-0.014,-0.003) | 0.001 (-0.001,0.003) |
| Female | 0.452 (0.009, 0.896) * | 2.28×10^-4^ (-0.010,0.009) | -0.014 (-0.024,-0.005)* | 3.25×10^-4^ (-0.003,0.002) |
| Linolelaidic acid |  |  |  |  |
| Male | 0.752 (0.287,1.217) * | -0.001 (-0.009,-0.007) | -0.005 (-0.014,-0.005) | 0.003 (0.001,0.006) |
| Female | 1.037 (0.565,1.508) * | -0.010 (-0.021,-0.000) | -0.020 (-0.031,-0.010)* | -0.002 (-0.005,0.001) |
| Sum TFAs |  |  |  |  |
| Male | 0.491 (0.059,0.922) * | 0.001 (-0.006,0.008) | -0.007 (-0.016,-0.002) | 0.002 (-0.001,0.004) |
| Female | 0.608 (0.125,1.090) * | 2.80×10^-4^ (-0.010,0.011) | -0.021 (-0.031,-0.011)* | -0.001 (-0.004,0.002) |

Note: FT3, free triiodothyronine; FT4, free thyroxine; TT4, total thyroxine; TT3, total triiodothyronine; CI, confidence interval; * P < 0.05; Model was adjusted by age, BMI, race category, UIC, alcohol category and smoking category.
